# Effect of Shiga Toxin and Its Subunits on Cytokine Induction in Different Cell Lines

**Published:** 2014

**Authors:** Neda Moazzezy, Mana Oloomi, Saeid Bouzari

**Affiliations:** 1 *Molecular Biology Unit, Pasteur Institute of Iran, Pasteur Ave. 13164 Tehran, Iran.*

**Keywords:** Cytokine, HeLa cell line, shiga toxin, THP1 cell line

## Abstract

Shiga toxins (Stxs) are bacterial virulence factors produced by *Shigella dysenteriae* serotype 1 and *Escherichia coli* strains. Stxs are critical factors for the development of diseases such as severe bloody diarrhea and hemolytic uremic syndrome. Additionally, Stxs trigger the secretion of pro- inflammatory cytokines and chemokines, particularly in monocytes or macrophages. The inflammatory cytokines result in the modulation of the immune system, local inflammations and enhancement of cytotoxicity. In this study, stimulation of the pro- inflammatory cytokines IL-1α, IL-1β, IL-6, IL-8, and TNF-α was assessed by recombinant Stx (rStx) and its subunits (rStxA and rStxB). Cytokines expression at mRNA level was investigated by Reverse Transcription-Polymerase Chain Reaction (RT-PCR) method in HeLa cells and THP1 monocyte/ macrophage cell lines. After incubation with rStx and its recombinant subunits, the expression of IL-1α, IL- 6 and IL- 8 mRNAs was strongly induced in HeLa cells. In HeLa cells, low expression of IL-1α mRNA was shown by rStxB induction. Furthermore, the expression of IL-1α and IL-1β mRNAs in undifferentiated THP1 cells was only induced by rStx. In differentiated THP1 cells, rStx and its recombinant subunits elicited the expression of IL-1α, IL-1β, IL-8 and IL- 6 mRNAs. On the other hand, expression of TNF-α mRNA was only induced by rStx. Based on the data, the profile of cytokine induction in response to the rStx, and its subunits differs depending on the cell types.

Shiga toxin (Stx) is a virulence factor produced by Enterohemorrhagic *E. coli* (EHEC) (1). It is also known as Verotoxin (VT) due to Vero cells’ sensitivity to Stxs (2). Stxs are essential for the development of severe bloody diarrhea and hemolytic- uremic syndrome (HUS) with worldwide prevalence, characterized by serious complications including renal failure, thrombocyto-penia and hemolytic anemia (3). Stxs have AB5 molecular structure, composed of one enzyma-tically active A subunit (32 kDa) and five identical B subunits (7.7 kDa each) that mediate the attachment of holotoxin (AB5) to neutral glycolipid receptor globotriaosylceramide (Gb3, or CD77) on the surface of host cells (4, 5). StxA and StxB subunits are separately secreted into the bacterial periplasm where they are noncovalently assembled into holotoxin (6). The A subunit with highly specific RNA *N*- glycosidase activity cleaves an adenine base of 28S ribosomal RNA (rRNA) of eukaryotic ribosomes (7). This is followed by the internalization of Stx into the target cell by endocytosis and transportation via the trans- Golgi network and Golgi apparatus to the endoplasmic reticulum (ER) and finally into the cytoplasm (8). Thus, elongation-factor-1-dependent aminoacyl tRNA binding is blocked by depurination reaction, thereby rendering ribosomes inactive for protein synthesis which leads to cell death in susceptible host cells (9).

Despite the fact that Stx has direct effect on protein synthesis (also termed the ribotoxic stress response) (9), its function on exacerbating cytotoxicity is not only restricted to ribotoxic stress. Stx also triggers programmed cell death (apoptosis) through various mechanisms in different cell types (10). It is crucial for the incidence of vascular lesions and tissue damage and subsequently translocation of the toxin into the bloodstream (11).

Following its entry into the cell, Stx stimulates signaling pathways via cellular stress- activated protein kinases. These include p38 mitogen- activated protein kinase/ERK (p38 MAPK/ERK) and c- Jun N- terminal kinase (JNK), whose pathways ultimately lead to the synthesis and the release of pro- inflammatory cytokines in susceptible cells such as human epithelial, endothelial and myeloid cells. The host innate immune response is regulated differentially by Stx through multiple mechanisms in both the transcriptional (mRNA expression) and trans-lational (protein secretion) levels of cytokine production (12). In comparison to many epithelial and endothelial cells in the kidney and the central nervous system that are originally sensitive to the cytotoxic action of Stx (13), primary human monocytes and macrophage- like cell lines are relatively resistant or insensitive, despite expressing Gb3 (14). Resistant cells generate soluble cytokines and chemokines such as tumor necrosis factor alpha (TNF-α) and interleukin-1β (IL-1β). These are essential agents contributing to the susceptibility of endothelial cells (as the primary target) to toxin. This is mediated by increasing the expression of Gb3 and different leukocyte adhesion molecules to elicit cytotoxicity of the toxin (15). The host response factors play an essential role in the development of hemorrhagic colitis and HUS by inducing vascular damage (16).

Cell culture studies have shown that epithelial cell lines like Caco-2 and HCT- 8 as well as human peripheral blood monocytes, primary human monocytes and macrophage- like cell lines can produce proinflammatory cytokines, especially the neutrophil chemoattractant IL- 8 in response to sub-lethal or nontoxic dose of Stxs *in vitro* (17, 18). On the other hand, it has been suggested that cytokine and chemokine expression is the result of Stx internalization inside the Gb3- negative cells (19). However, retrograde transport pathway from Golgi to endoplasmic reticulum (ER) is necessary for Stx cytotoxicity at least in some cell types (20, 21). Thus eukaryotic cell lines with different Stx sensitivity (high and low content of Gb3) could synthesize pro- inflammatory cytokines.

In the present study, the expression of pro- inflammatory cytokines IL- 1α, IL-1β, IL-6, IL-8, and TNF- α was assessed by treatment with rStx, rStxA and rStxB subunits in HeLa and THP1 monocyte/macrophage cell lines. 

## Materials and Methods

**Holotoxin and its subunits**

Stx as holotoxin and its subunits were obtained from previous study (22).The obtained crude toxins were applied to Endotrap (profos AG) (endotoxin removal systems in order to remove LPS content). The samples were sterilized by filtering through low protein binding filter (Miller®- GV 0.22 µm filter unit, Millipore) and the protein concentration was estimated using Bradford method by measuring absorbance at 595 nm with bovine serum albumin as control (Protein assay Kit, Bio-Rad).

**Cell culture**

The human myelogenous leukemia cell line THP-1 and cervical cancer HeLa cells were purchased from (National Cell Bank, Pasteur Institute of Iran) and cultured in RPMI-1640 medium (Biosera) supplemented with penicillin (100U/ml), streptomycin (100µg/ml ) and 10% fetal bovine serum. Cells were grown and maintained at 37°C in 5% CO2 in a humidified incubator.

**Macrophage differentiation**

The mature macrophage-like cells were induced by treating THP-1 cells (10^6^ cells/ ml) with 50 ng/ ml phorbol 12-myristate 13 acetate (PMA) for 72 hours. Plastic- adherent cells were washed twice with cold, sterile Dulbecco phosphate- buffered saline (PBSRPMI 1640 lacking PMA but containing 10% FBS, penicillin (100 U/ ml) and streptomycin (100 µg/ ml). The medium was then changed every 24 hours for 3 additional days and the experiment was continued for four days after the removal of PMA.

**Stimulation of cells**

Confluent HeLa cells and other cell lines were treated with recombinant Stx, StxA and StxB subunits (10 µg/ ml) separately for 24 hours. The recombinant Stx dose used in this experiment displays approximately 50% cytotoxicity for 10^6^ HeLa cells (23). In each experiment, cell lines without treatment were used as negative control.

**Isolation and analysis of total cellular RNA**

Total cellular RNA was isolated according to FastPure ™RNA Kit (TaKaRa). The FastPure RNA Kit allows simple and quick extraction of highly pure total RNA without DNase treatment from cultured cells via centrifugation. The first step was the preparation of lysate via homogenization of cells (about 1 x 10^6 ^cells) using supplied lysis and solubilization buffers. Then the lysate was placed in a cartridge containing a polymeric filter with affinity for nucleic acids. Total RNA was collected from the cartridge by microcentrifugation. The extracted RNA was assessed by spectrophotometer and the concentration of RNA was determined by measuring absorbance at 260 nm (A260). RNA purity was analyzed by the ratio between absorbance values at 260 and 280 nm. Total RNA extraction was assessed by agarose gel electrophoresis and ethidium bromide staining. Then, the extracted purified RNA was applied to Reverse Transcription- Polymerase Chain Reaction (RT-PCR).

**Detection of pro- inflammatory cytokine mRNA by reverse transcription-** **PCR** **(RT-** **PCR** **)**

Total extracted RNA was amplified by one step AccuPower® RT- PCR Premix (BioNEER) Kit. All components necessary for cDNA synthesis and amplification were provided in one tube. The primers used in this survey are shown in Table 1.

**Table 1:** Cytokine primers and size of RT-PCR products used in this study

Cytokine	F primers (5'-3')	R primers (5'-3')	Size of PCR product (bp)
IL-8	GTGTGAAGGTGCAGTTTTGC	GCAGTGTGGTCCACTCTC AA	126
IL-6	CTTCTCCACAAGCGCCTT C	GCGGCTACATCTTTGGAATC	113
IL-1α	CAGTGCTGCTGAAGGAGATG	AAGTTTGGATGGGCAACTGA	123
IL-1β	CAGTGGCAATGAGGATGACT	TCGGAGATTCGTAGCTGGAT	116
TNF-α	TCAGATCATCTTCTCGAACC	CAGATAGATGGGCTCATACC	358
GAPDH	GGTCGGAGTCAACGGATTTG	ATGAGCCCCAGCCTTCTCCAT	318

GAPDH, glyceraldehyde-3-phosphate dehydrogenase; IL, interleukin; TNF-α, tumor necrosis factor-α

The quality of RNA isolates was verified by the amplification of GAPDH (glyceraldehyde-3-phosphate dehydrogenase). Presence of GAPDH mRNA (31.2pg) was taken as an internal control. In this experiment, extracted RNA (1-2 µg) was used with the same amount of GAPDH RT- PCR product in order to obtain the same sensitivity for each cytokine. The extracted RNA and the reverse primer were mixed and incubated at 70ºC for 5 min. The incubated mixture and the forward primer were then transferred to a premix tube. The cDNA synthesis was performed at 42ºC for 60 min and at 94ºC for 5 min. PCR cycles were carried out as follows: 94ºC for 60 sec, 54ºC for 30 sec and 72ºC for 60 sec with a final 10 min extension at 72ºC. For all of the genes tested in this study (including the ubiquitous housekeeping gene GAPDH), 30 cycles of PCR was found optimal. The aliquots of PCR products hence obtained were visualized after electrophoresis on 2% agarose gel under UV in a gel documentation system and quantified by Lab Gel Doc Image software.

**Statistical Analysis**

The results were expressed as the Mean ±SEM for the number of experiments. The statistical significance was compared between each treatment group and the control by Student’s t test. The analyses were performed using SPSS software version 18.

## Results

**Cytokine mRNAs expression in HeLa cells**

**Fig. 1:**
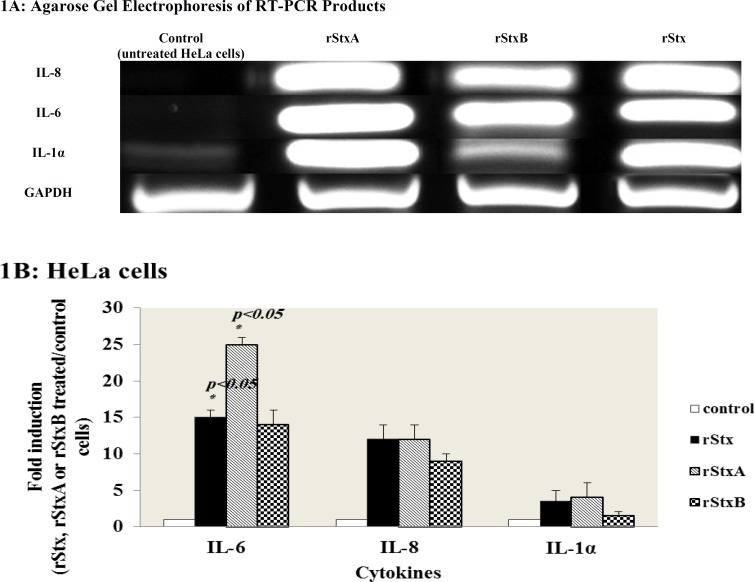
Induction of cytokine mRNAs by rStx and its subunits in HeLa cells (A): Expression of cytokine mRNAs detected by RT-PCR, using total RNA extracted from HeLa cells treated with rStx, rStxA and rStxB for 24 h. GAPDH is included as an internal control for total RNA. Each sample was subjected to electrophoresis on a 2% agarose gel and visualized by ethidium bromide staining. (B) Quantification of Cytokines expression analysis in was performed using Gel Doc software. Ratios (between Stx-induced cytokine levels and control levels that had not been treated with rStx, rStxA or rStxB) shown are the mean ± standard error of means for duplicate experiments. (p*-*values were generated with a two-tailed Student *t *test).

HeLa cells were treated with sub-lethal concentration of rStx, rStxA and rStxB subunits for 24 hours and then total cellular RNAs were extracted from treated and untreated cells and the expression of inflammatory cytokine mRNAs IL-6, IL-8, IL-1α, IL-1β, and TNF-α was examined by RT-PCR (Figure 1A). In Figure 1B, a ratio between the expression of the mRNA of interest in rStx and its recombinant subunits, rStxA- and rStxB- treated HeLa cells and that in control cells is shown. IL- 6 mRNA inductions were observed for rStxA (25.00± 1.00 fold increase) rStxB (14.00± 2.00 fold increase) and rStx (15.00± 1.00 fold increase). The C-X-C chemokine IL- 8 mRNA inductions were observed for rStx and rStxA (12.00± 2.00 fold increase) and rStxB (9.00± 1.00 fold increase). IL-1α mRNA inductions were observed 3.50± 1.50 and 4.00± 2.00 fold increase by rStxA and rStx respectively at 24 hours. In contrast, rStxB did not have any remarkable effect on IL-1α mRNA expression. Finally, the treatment of HeLa cells with rStx, rStxA or rStxB did not evoke the elevated IL-1β or TNF-α expression (data not shown).

**Cytokine mRNAs expression in THP1 monocyte cells**

**Fig. 2:**
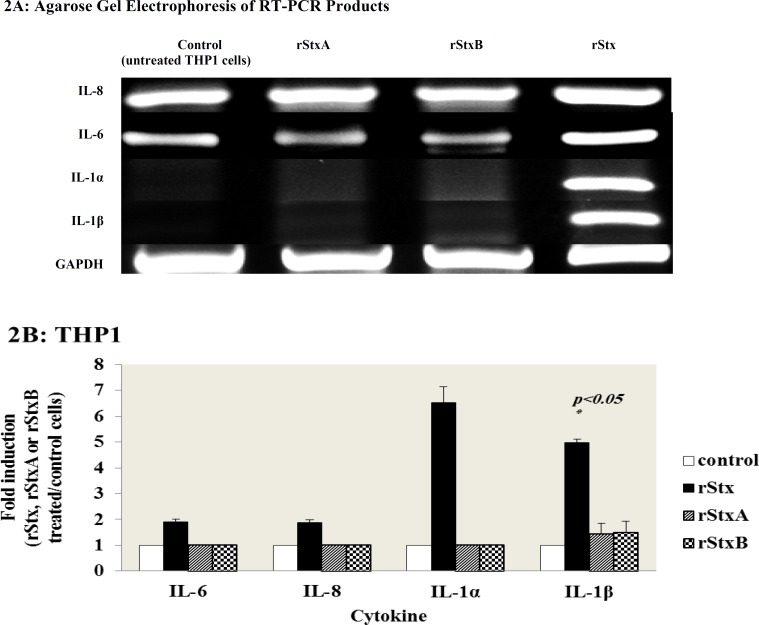
Induction of cytokine mRNAs by rStx and its subunits in THP1 cells (A) Expression of cytokine mRNAs detected by RT-PCR, using total RNA extracted from THP1cells treated with rStx, rStxA and rStxB for 24 h. GAPDH is included as an internal control for total RNA. Each sample was subjected to electrophoresis on a 2% agarose gel and visualized by ethidium bromide staining. (B) Quantification of Cytokines expression analysis was performed using Gel Doc software. Ratios (between Stx-induced cytokine levels and control levels that had not been treated with rStx, rStxA or rStxB) shown are the mean ± standard error of means for duplicate experiments. (p- values were generated with a two-tailed Student *t *test).

Earlier investigations have indicated that monocytic cells are sensitive to the Stx treatment and they could synthesize the inflammatory cytokines (13). Total cellular RNAs from treated and untreated THP1 cells were extracted and cells were analyzed by RT-PCR to investigate the expression of IL-1α, IL-1β, IL-6, IL-8 and TNF-α transcript after 24 hours treatment with sub-lethal concentration of rStx, rStxA, and rStxB (Figure 2A).

As shown in Figure 2B, IL-6, IL-8, IL-1α and IL-1β mRNA inductions were observed for rStx 1.90± 0.10, 1.88± 0.11, 6.52± 0.62 and 4.98± 0.13 fold increases, respectively, where numbers expressed are Mean±SEM expression levels between the two different donors. However, RT-PCR analysis did not show any expression of IL-6, IL-8, IL-1α and IL-1β cytokines in response to recombinant subunits of Stx treatment in THP1 cells.

In addition, no induction of cytokine mRNA of TNF-α was observed by the recombinant Stx, StxA or StxB (data not shown).

**Cytokine mRNAs expression in PMA-treated THP1 cells**

**Fig. 3:**
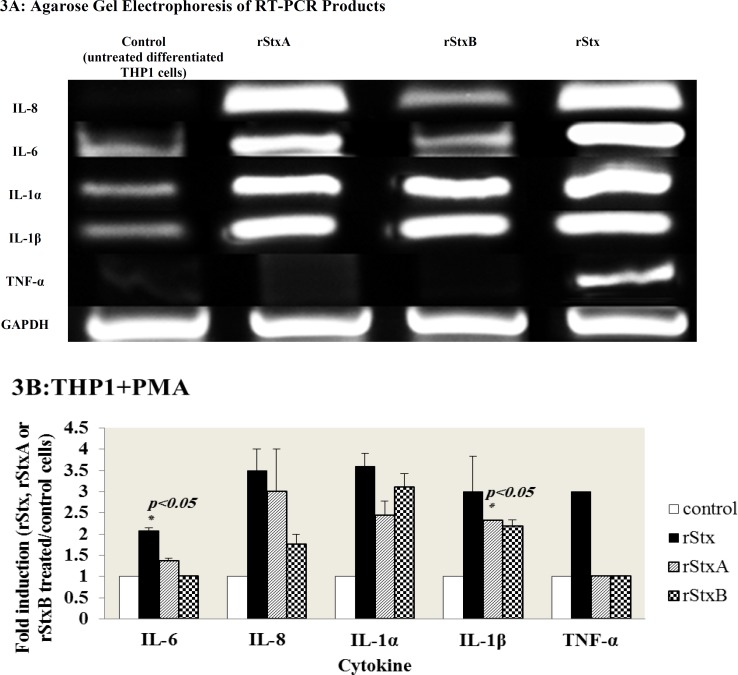
Induction of cytokine mRNAs by rStx and its subunits in differentiated THP1 cells (A) Expression of cytokine mRNAs detected by RT-PCR, using total RNA extracted from differentiated THP1 cells treated with rStx, rStxA and rStxB for 24 h. GAPDH is included as an internal control for total RNA. Each sample was subjected to electrophoresis on a 2% agarose gel and visualized by ethidium bromide staining. (B) Quantification of Cytokines expression analysis was performed using Gel Doc software. Ratios (between Stx-induced cytokine levels and control levels that had not been treated with rStx, rStxA or rStxB) shown are the mean ± standard error of means for duplicate experiments. (p-values were generated with a two-tailed Student *t *test).

Optimal stimulation of cytokine expression by 

human monocyte cell lines could be induced after cellular differentiation; thus we used PMA to differentiate THP1 monocyte cell lines (24). Differentiated THP1 cells were also incubated with sub-lethal concentration of rStx, rStxA and rStxB for 24 hours. Total cellular RNAs from treated and untreated cells were extracted after incubation period and mRNA level for inflammatory cytokines was determined by RT-PCR (Figure 3A).

As shown in Figure 3B, IL-6 mRNA inductions were observed for rStxA (1.36 ± 0.06 fold increase) and rStx (2.07± 0.07 fold increase) but no induction of cytokine mRNA of IL-6 was observed by the rStxB. The chemokine IL-8 transcript inductions were observed for rStx, rStxA, and rStxB (3.50± 0.50, 3.00± 1.00, and 1.75± 0.25 fold increases, respectively). IL-1α mRNA expression was increased 3.60± 0.30 fold by rStx, 2.43± 0.35 fold by rStxA, and 3.10± 0.33 fold by rStxB. The rStx, rStxA and rStxB have enhanced IL-1β induction 3.00± 0.84, 2.31± 0.01, and 2.18± 0.16 fold increases, respectively. Finally, TNF-α mRNA induction was enhanced just by rStx (3-fold increase). Numbers expressed are Mean± SEM expression levels between the two different donors.

## Discussion

Shiga toxins (Stxs) are multifunctional proteins that in addition to protein synthesis inhibition are involved in multiple cellular effects such as enhancement of cytotoxicity through cytokine synthesis via triggering signaling pathway in various eukaryotic cell types (25).

In the present study, the effects of sub-lethal concentration of rStx (holotoxin), rStxA subunit with *N*-glycosidase activity and rStxB subunit that mediates toxin binding to cells was assessed. The expression of pro-inflammatory cytokines mRNA in different cell lines with different levels of susceptibility to toxin-binding (different levels of Gb3 expression) was evaluated.

Epithelial cells are being the first barrier of defense against bacteria; earlier researchers have been focused on these cell lines. Expression of chemokine IL-8 in intestinal epithelial T-84 (Gb^3-^) cells was shown with Stx and StxB treatments (26). In HCT-8 and caco2 (Gb^3+^) cells IL-8 expression was only observed by holotoxin (26, 27). Thus, both holotoxin and stxA could induce pro-inflammatory cytokines in epithelial cell lines. Being rich in Gb3 receptors on the surface and very sensitive to Stxs, HeLa cells might behave differently from other epithelial cell lines via cytokine induction through treatment with Stx (28). In another study, HeLa cells were incubated with rStx or rStxA for 24 hours, resulting in the induction of expression of the pro-inflammatory cytokines, IL-1α and IL- 6 which play a well-documented role in the activation of the mucosal inflammatory response. The expression of C-X-C chemokine IL-8, a potent chemoattractant for recruitment of these cells into the infected epithelium was also detected (29). In addition, our results indicated that rStxB-treated HeLa cells also induce pro- inflammatory mRNAs, similar to previous works on T- 84 human colon cancer cell line (26).

In contrast, Yamasaki et al*.* showed that there is not a direct link between cytokine inducing activity and Gb3 mediated signaling in human colon Caco- 2 cells at the mRNA level by mutant Stx which lacked *N*-glycosidase activity (29).

Nonetheless, Nakagawa et al. showed that expression of Stx1B in cytoplasm of HeLa cells may induce pro-inflammatory cytokines by activating caspase-1 (30). Thus, it is considerable that different epithelial cells do not present the same or similar behavior to StxB subunit. In these experiments, higher levels of mRNA induction in HeLa cells for IL-8, especially IL-6 and IL-1α by sub-lethal amounts of rStxA in comparison to rStx or rStxB support the concept that rStxA can be a more potent inducer of pro inflammatory cytokines. The expression of IL-1α mRNA in HeLa cells just by rStxA and rStx indicates that the induction of IL-1α can be solely attributed to RNA *N*-glycosidase activity. Induction of IL-8 and IL-6 by rStxB suggests that Gb3-mediated signaling pathway in HeLa cells may induce cytokines.

In the present study on highly toxin sensitive cells, in contrast to IL-1α, IL-8 and IL-6, induction of TNF-α or IL-1β mRNA was not detected by RT-PCR, similarly to the observations of Fujii et al. who demonstrated a rapid cytotoxicity and no expression of Fas in HeLa cells (31).

Monocyte and macrophage cells are the cellular components of innate immunity that have the major role in the pathogenesis of hemolytic uremic syndrome via production of proinflammatory cytokines including soluble cytokines TNF- α and IL-1β. These inflammatory mediators up-regulate Gb3 expression receptors on human vascular endothelial cells in response to Stxs that may elicit cytotoxicity and vascular damage (16). As we expected about the differentiation (with a loss of susceptibility to Stxs) and effects on the level of cytokine induction (13), our data revealed that undifferentiated THP1 cells respond differently to treatment with rStx and its recombinant subunits compared to PMA treated THP1 cells. It was shown that PMA treated THP1 cells are more capable of producing proinflammatory and endogenous cytokines mRNAs compared to THP1 monocytic cells in response to Stx and especially its subunits. On the other hand, cytokine expression by Stxs subunits was only stimulated in differentiated THP1 monocyte/ macrophage cell lines. Moreover, regarding TNF-α, the expression has only been observed in differentiated THP1 cells exclusively by rStx holotoxin. Both toxin attachment to target cell and its enzymatic activity may be required for activating cellular-stress signaling response and consequently the expression of TNF-α in differentiated THP1 (13). TNF-α transcripts induced by Stxs appeared relatively stable over a 4 to 12 h time period (14). It seems that the incubation time in our experiment was not adequate for the detection of TNF-α by recombinant subunits, especially rStxA. In contrast to THP1 monocytic cells, recombinant StxA and StxB induced significant expression of IL-8, IL-6 and especially IL-1α and IL-1β mRNAs. So these data suggest that differentiation of THP1 cells by PMA can also induce the expression of proinflammatory cytokines by recombinant subunits of shiga toxins.

Our cell culture studies are consistent with the hypothesis that the treatment of THP1 cells with differentiation agents such as PMA can decrease Gb3 receptor expression and consequently toxin sensitivity of the cells is reduced. So these cells become more resistant to the cytotoxic function of Stxs and acquire the ability to express more cytokines particularly IL-1β and TNF-α which significantly increase Gb3 receptors on endothelial cells and exacerbate vascular endothelial cell damage by inhibition of cellular functions (32).

The data suggest that in response to Stxs, the expression of pro-inflammatory cytokines modulates in HeLa, THP1 and PMA- stimulated THP1 cell lines, but between these cell lines, monocyte/ macrophage THP1 cells can have a pivotal role to induce TNF-α and IL-1β cytokines as factors increasing cytotoxicity. rStxB has particular applications in biomedical research such as tumor targeting and immunotherapy. A significant implication of this study suggests that rstxB can act as the holotoxin (rStx) in cytokine expression in HeLa and differentiated THP1 cells. However, the effect of these subunits on monocytic or differentiated THP1 remains controversial. Thus, the profile of cytokine induction in response to the Stxs varies depending on the cell types. On the other hand, it appears that recombinant subunit of Stx activates distinct signaling pathways to induce the cytokine expression. In conclusion, to further understand the effects of Shiga toxin and its subunits on cytokine induction in different cell lines, more research on the underlying molecular mechanisms is suggested. 

## Conflicts of interest

The authors declared no conflict of interest.
